# Identifying targetable alterations predictive of distant progression in glioblastoma patients undergoing standard therapy

**DOI:** 10.1093/noajnl/vdaf092

**Published:** 2025-05-07

**Authors:** Shivani Chiranth, Vincent Fougner, Ib Jarle Christensen, Anouk Kirsten Trip, Terkel Christiansen, Dorte Schou Nørøxe, Christina Westmose Yde, Anne Kiil Berthelsen, Benedikte Hasselbalch, Ulrik Lassen, Hans Skovgaard Poulsen, Thomas Urup

**Affiliations:** The DCCC Brain Tumor Center, Rigshospitalet, Copenhagen, Denmark; The DCCC Brain Tumor Center, Rigshospitalet, Copenhagen, Denmark; Department of Oncology, Rigshospitalet, Copenhagen, Denmark; The DCCC Brain Tumor Center, Rigshospitalet, Copenhagen, Denmark; The DCCC Brain Tumor Center, Rigshospitalet, Copenhagen, Denmark; Danish Centre for Particle Therapy, Aarhus University Hospital, Aarhus, Denmark; The DCCC Brain Tumor Center, Rigshospitalet, Copenhagen, Denmark; Department of Oncology, Rigshospitalet, Copenhagen, Denmark; The DCCC Brain Tumor Center, Rigshospitalet, Copenhagen, Denmark; Department of Oncology, Rigshospitalet, Copenhagen, Denmark; Center for Genomic Medicine, Rigshospitalet, Copenhagen, Denmark; Department of Oncology, Rigshospitalet, Copenhagen, Denmark; The DCCC Brain Tumor Center, Rigshospitalet, Copenhagen, Denmark; Department of Oncology, Rigshospitalet, Copenhagen, Denmark; The DCCC Brain Tumor Center, Rigshospitalet, Copenhagen, Denmark; Department of Oncology, Rigshospitalet, Copenhagen, Denmark; The DCCC Brain Tumor Center, Rigshospitalet, Copenhagen, Denmark; The DCCC Brain Tumor Center, Rigshospitalet, Copenhagen, Denmark; Department of Oncology, Rigshospitalet, Copenhagen, Denmark

**Keywords:** distant progression, progression pattern, IDHwt glioblastoma, glioma migration, invasion

## Abstract

**Background:**

Infiltrative growth is a hallmark of glioblastoma (GBM) and is a major factor in therapeutic failure. Distant progression is a surrogate marker for infiltrative growth and genetic variants predictive of distant progression may serve as novel treatment targets. The aim was to identify clinical, molecular, radiographic, and genetic factors associated with distant progression in GBM patients.

**Methods:**

From our prospective database, all consecutive GBM *IDH* wild type patients receiving standard therapy (Stupp regimen) from 2016 to 2021 at Rigshospitalet (Denmark) were included. Distant progression was defined as a new contrast-enhancing lesion > 2 cm from the initial lesion. Clinical, molecular, radiographic, and genomic covariates were assessed for association with time to distant progression using Cox analysis.

**Results:**

This single-center study included 353 patients, of whom 303 patients had radiographic progression. Distant progression was found in 66 patients (22%) and was associated with poor post-progression survival (*P* < .001). Unmethylated *MGMT* (hazard ratio [HR]: 2.54, 95% confidence interval [CI]: 1.62–3.97, *P* < .001), and multicentric disease at diagnosis (HR: 2.32, 95% CI: 1.28–4.20, *P* = .005) were associated with a shorter time to distant progression. In patients with a genomic tumor profile (*n* = 204), the *NF1* gene alteration was identified as an independent predictor of distant progression (HR: 3.48, 95% CI: 1.48–8.21, *P* = .004) and poor survival.

**Conclusion:**

Distant progression is an aggressive progression pattern associated with a poor prognosis. Unmethylated *MGMT,* multicentric disease, and *NF1* alteration independently predict distant progression. *NF1* alteration may serve as a predictive biomarker for targeted treatment.

**Key Points**

- Distant progression is associated with a poor prognosis.
- Unmethylated *MGMT,* multicentric disease, and *NF1* alteration predict distant progression.
- *NF1* alteration is a prognostic factor and may serve as a predictive factor for targeted treatment.

**Importance of the Study**

Glioblastoma is characterized by infiltration into neighboring brain parenchyma, contributing to treatment failure, neurological deterioration, and early mortality. Distant progression, defined as tumor progression located more than 2 cm from the initial tumor, is a marker for infiltrative growth. By combining genomic tumor profiling and clinical data, we aim to identify predictors of distant progression, including targetable drivers of glioma migration. Our study confirmed that distant progression after standard therapy is an aggressive progression pattern associated with a poor prognosis. Three independent predictors, unmethylated *MGMT,* multicentric disease, and *NF1* alteration, were found to be associated with a shorter time to distant progression. We hypothesize that *NF1* alteration serves as a predictive biomarker for targeted treatment of migratory growth.

Glioblastoma (GBM) is the most common malignant primary brain tumor in adults. For patients receiving standard treatment, including maximal safe surgical resection followed by long-course radiation therapy with concomitant and adjuvant temozolomide, intracranial tumor progression is almost inevitable, and the prognosis is poor with a median overall survival of 15–18 months.^1–3^

The infiltrative nature of GBM is one of the major contributing factors to treatment failure. GBM cells migrate along the perivascular space and white matter tracts within the brain parenchyma.^4^ Consequently, GBMs can progress both at the site of the initial tumor (local progression) and at distant sites within the brain (distant progression), including the contralateral hemisphere or the brainstem. Previous studies have shown that distant progression, identified by magnetic resonance imaging (MRI) in about 20% of patients, is associated with a significantly worse prognosis.^5–8^ Accordingly, we hypothesize that distant progressing tumors reflect a more migratory subtype and that improved characterization of this progression pattern can lead to the identification of targetable drivers of glioma migration.

Progression patterns have been studied in several studies. However, no consistent baseline biomarkers predictive of distant progression have been identified to date.^5,8–10^ By combining genomic tumor profiling and clinical data, we aim to achieve a deeper understanding of the factors driving tumor cell migration and identify biomarkers predictive of a shorter time to distant progression. In a prospectively registered cohort of consecutive *IDH*-wildtype (*IDH*wt) GBM patients treated with standard therapy, we retrospectively reviewed radiological data to (1) characterize the progression pattern and (2) identify biological factors that drive distant progression. Lastly, our cohort of patients with a genomic tumor profile was analyzed to (3) identify targetable genomic drivers of distant progression.

## Materials and Methods

### Patient Population

This study included all GBM *IDH*wt patients treated with standard therapy, defined as maximal safe surgical resection and long-course chemoradiotherapy (60 Gy in 30 fractions combined with temozolomide), at Rigshospitalet, Denmark, from January 2016 to December 2021. All patients were prospectively registered in our clinical database. Patients were eligible to receive standard treatment if they had histologically verified GBM, ECOG (Eastern Cooperative Oncology Group) performance status (PS) ≤ 2, and sufficient renal, hepatic, and hematologic function.^3^ The cutoff date for the study was December 2022.

All patients were included in a clinical cohort, and their tumor radiology was retrospectively reviewed to assess baseline characteristics and progression patterns. In the study period, patients were offered a genomic profile of the diagnostic tumor tissue, and those who consented to genetic analysis were included in the genomic subcohort.^11,12^

The study was performed in accordance with the Declaration of Helsinki and Danish legislation and has been approved by the local ethical committee (H-19054690) and the Danish National Ethics Committee (H-21023801).

### Treatment and Follow-Up

All included patients were at initial diagnosis evaluated for surgery. In cases where surgical resection was not performed, a biopsy was taken to confirm the diagnosis. Subsequently, all patients were planned for radiotherapy (60 Gy in 30 fractions) with concomitant temozolomide followed by intended 6 cycles of adjuvant temozolomide as described in more detail by Abedi et al.^3^

For patients in the genomic cohort, tumor tissue was analyzed by RNA sequencing, whole exome sequencing (WES), panel sequencing, or more recently whole genome sequencing (WGS)^11–13^

Contrast and non-contrast MRI was performed at the time of debut, postoperatively (within 72 hours), 3 months after radiation therapy, and every 3 months until death or termination of follow-up. Treatment response was evaluated in accordance with the Response Assessment in Neuro-Oncology (RANO) criteria.^14^

In case of progression, patients were assessed for re-operation, and/or second-line treatment with temozolomide, lomustine, bevacizumab combination therapy with lomustine or irinotecan, or available experimental protocols.

### Candidate Factors

Several candidate clinical, molecular, radiological, and genetic factors were selected to be screened for association with distant progression. These factors are defined in the following section.

#### Clinical factors.

—The clinical factors included age, gender, ECOG PS, and resection vs biopsy at diagnosis. Corticosteroid use defined as patients receiving > 10 mg/day of prednisolone at the start of concomitant radiotherapy and temozolomide treatment was also registered.

#### Biomarker analysis.

—As described in Abedi et al, formalin-fixed paraffin-embedded tumor tissue was analyzed by immunohistochemistry (IHC) to determine p53 protein expression and *IDH1* mutation status. In more recent cases, *IDH* negative patients determined by IHC, who were younger than 55 years of age were sequenced for *IDH1/2* mutation status according to the 2016 WHO classification system.^15^*MGMT* status was determined by examining *MGMT* promoter methylation by pyrosequencing (Qiagen) using a cutoff of 10% mean *MGMT* methylation.^3,16^

#### Genomic analysis.

—Patients in the genomic cohort received genomic analysis in the form of WES, WGS, or panel sequencing with Trusight oncology (TSO-500) and RNAseq on tumor tissue.^11^ Tumor tissue from patients with debut of disease prior to 2021, underwent WES analysis. Starting in January 2021, WGS was used to analyze frozen tissue, and TSO-500 was employed for paraffin-embedded tissue. The genomic analysis was performed at the Department of Genomic Medicine, Rigshospitalet, and pathogenic and likely pathogenic alterations from the genomic reports were registered and analyzed.^12,13^ Genetic alterations were either (1) mutations, defined as pathogenic or likely pathogenic single-nucleotide variants, insertions/deletions, and fusions; (2) amplifications; and (3) biallelic deletions. Germline variants for genes were tested using a panel since March 2021. The panel included the following genes: *BRCA1, BRCA2, ATM, MLH1, MSH2, MSH3, MLH2, MLH3, MSH6, PMS2, PALB2, RAD51C, RAD51D*, and *MBD4*. Prior to this, germline alterations were not assessed systematically.

Candidate genetic factors comprised (1) gene alterations present in more than 5% of the analyzed samples (dichotomized), and (2) Altered GBM signaling pathways present in more than 5% of analyzed cases (the pathways were dichotomized as either altered or not based on alterations of one or more genes belonging to it as defined by Sanchez-Vega et al.).^17^

#### Baseline radiological factors.

—Radiological tumor characteristics such as multicentric disease, the tumor’s anatomical location in relation to brain lobes and the ventricle system, as well as the extent of surgical resection were reviewed. Multicentric disease was defined as 2 or more contrast-enhancing lesions measured to be more than 1 cm apart without a connecting T2-weighted fluid-attenuated inversion recovery (T2 FLAIR) hyperintense signal.^18^ The tumor was classified as involving the subventricular zone if the contrast-enhancing lesion was in contact with the ventricle system in the preoperative MRI.

The extent of surgical resection was evaluated using post-operative MRI and was categorized as (1) gross total resection, defined as no residual contrast-enhancing tumor, (2) near total resection, defined as a non-measurable contrast-enhancing tumor, and (3) subtotal resection, defined as measurable residual tumor according to the RANO criteria.^14,19^

### Definition of Progression Pattern

The progression pattern, categorized as distant progression or local progression, was evaluated by 2 investigators (S.C. and T.U.) by reviewing the MRI at the time of progression. In case of disagreement, the images were evaluated by an experienced neuro-oncology radiologist (A.K.B.) to reach a consensus. Distant progression was defined as the presence of a new contrast-enhancing lesion more than 2 cm from the primary contrast-enhancing tumor or resection cavity. The cases in which the recurrent tumor was in contact with the primary tumor or where a new contrast-enhancing lesion was located within 2 cm from the primary tumor or resection cavity were classified as local progression.^10^ The distance between 2 lesions was measured as the shortest distance between their margins. In the instance of multiple lesions or both local and distant progression occurring simultaneously, the progression pattern was classified as distant, and the distance was measured to the most remote of the lesions. For the distantly progressing tumors, additional radiological factors like location or the presence of a T2 FLAIR signal connecting the lesion to the operation cavity were noted. Additionally, in those patients who only had local progressions after standard treatment, the subsequent follow-up MRI scans were analyzed to determine if they experienced distant progression at a later time. These patients were subsequently categorized as distant progression in a second multivariate model.

### Statistical Analysis

Comparison analysis was performed using the chi-square test and Mann–Whitney *U* test.

Survival probabilities were estimated with the Kaplan–Meier method. Progression-free survival (PFS) was defined as the time from diagnosis to progression or death of any cause, and overall survival (OS) as the time from diagnosis to death of any cause. The post-progression survival was the time from first progression to death of any cause and this was used in a landmark analysis of the progression pattern. Patients who did not reach the survival endpoint were censored.

The Cox proportional hazards model was used for modeling time to distant progression and patients with local progression were censored. Results are presented as hazard ratios (HR) with 95% confidence intervals (CIs) and the concordance index (c-index) is presented as a measure of discrimination. Assessment of the model assumptions was done using martingale residuals. Factors associated with time to distant progression with *P*-values below 0.30 in univariate analysis were considered for multivariate analysis adjusted for known independent prognostic factors. Ten-fold cross-validation was used for internal validation. The analysis was performed with and without death and clinical progression prior to MRI evaluation as a competing risk. A *P*-value of <.05 was considered statistically significant. The statistical analyses were performed using IBM SPSS statistics (version 29), SAS (version 9.4, SAS institute, Cary, N.C., USA) and R (version 4.3.1, and “package” RMS)

## Results

### Patients

This study included a total of 353 consecutive patients with GBM (WHO grade 4 *IDH*wt) treated with standard therapy (Table 1). There was an overweight of male patients (62%) with a median age of 59 years (range: 17–77 years). Most of the tumors were localized in the frontal lobe (28%), temporal lobe (24%), or were overlapping more than 1 lobe (27%) and 153 patients (43%) were found to have tumors involving the subventricular zone. Multicentric disease was found in 45 patients (13%).

**Table 1.** Patient Characteristics of Clinical Cohort (*n *= 353)

Recurrence pattern	Total, *n = *353	Local^a^, *n *= 237	Distant^a^, *n* = 66	*P*-value
Median age, years (range)	59 (17–77)	59 (23–75)	57 (17–73)	.20
Gender, *n* (%)				.25
Female	134 (38)	90 (38)	20 (30)	
Male	219 (62)	147 (62)	46 (70)	
ECOG performance status, *n* (%)				.57
0	208 (60)	139 (60)	43 (66)	
1	124 (35)	85 (36)	19 (29)	
2	17 (5)	10 (4)	3 (5)	
Missing	4	3	1	
Subventricular zone involvement, *n* (%)				.07
yes	153 (43)	105 (44)	21 (32)	
no	200 (57)	132 (56)	45 (68)	
Multicentric disease, *n* (%)				.96
Yes	45 (13)	28 (12)	8 (12)	
No	307 (87)	208 (88)	58 (88)	
Missing	1	1		
Tumor localization, *n* (%)				.19
Frontal lobe	99 (28)	67 (28)	20 (30)	
Temporal lobe	86 (24)	60 (25)	7 (11)	
Parietal lobe	40 (11)	25 (11)	11 (17)	
Occipital lobe	9 (3)	7 (3)	2 (3)	
Overlapping lobes	95 (27)	65 (27)	22 (33)	
Not defined	24 (7)	13 (6)	4 (6)	
Corticosteroid use^b^, *n* (%)				.44
Yes	161 (46)	104 (44)	25 (39)	
No	186 (54)	130 (56)	39 (61)	
Missing	6	3	2	
Resection, *n* (%)				0.98
Biopsy	65 (19)	39 (17)	11 (17)	
Resection	286 (81)	197 (83)	55 (83)	
Missing	2	1		
Extent of surgical resection, *n* (%)				.20
Gross total resection	98 (38)	66 (37)	25 (51)	
Near total resection	88 (35)	62 (35)	12 (24)	
Subtotal resection	68 (27)	49 (28)	12 (25)	
Missing	99	60	17	
*MGMT* status, *n* (%)				.72
Methylated	148 (42)	92 (39)	24 (36)	
Unmethylated	205 (58)	145 (61)	42 (64)	
p53, *n* (%)				**.02**
Positive	215 (68)	149 (70)	32 (53)	
Negative	103 (32)	64 (30)	28 (47)	
Missing	35	24	6	
Median PFS, (95% CI)	7.5 (6.8–8.2)	6.6 (5.5–7.7)	8.8 (7.6–10.0)	.10
Median OS, (95% CI)	17.2 (15.8–18.7)	NA	NA	
Resection at recurrence, *n* (%)				**<.001**
Yes	115 (35)	102 (44)	12 (19)	
No	215 (65)	131 (56)	53 (81)	
Missing	23	4	1	
Second-line treatment, *n* (%)				.13
Yes	240 (74)	191 (83)	47 (75)	
No	84 (26)	39 (17)	16 (25)	
Missing	29	7	3	

^a^Only for patients with radiographically confirmed progression (303).

^b^Prednisolone dose > 10 mg/day at initiation of concomitant treatment.

Abbreviations: *MGMT*, O6-methylguanine-DNA methyltransferase; PFS, progression-free survival; OS: overall survival. P values < 0.05 are considered significant and are made bold.

Most patients underwent surgical resection (*n* = 286, 81%). A post-operative MRI was performed in 254 patients of whom 98 underwent gross total resection (38%), 88 underwent near total resection (35%), and 68 underwent subtotal resection (27%). A diagnostic stereotactic biopsy was performed in 65 patients who did not undergo surgical resection (19%). 148 patients (42%) had methylated promoter of the *MGMT* gene, and 215 patients (68%) had protein expression of p53. Almost all patients (95%) had PS 0–1, and more than half (54%) were tapered off corticosteroids at the time of initiation of concomitant chemoradiotherapy.

As shown in Supplementary Figure S1, a total of 332 patients (94%) had tumor progression, among whom 303 patients (86%) had radiographic progression, and the remaining 29 patients had clinical progression without a confirmatory MR. At the time of first progression, 114 patients (34%) were surgically resected, and 240 patients (74%) received second-line treatment. For patients without progression, the minimum follow-up was 11.9 months, and no patients were lost to follow-up. The median OS was 17.2 months (95% CI: 15.8–18.6 months) and the median PFS was 7.5 months (95% CI: 6.8–8.2 months).

Out of the total study cohort, 204 patients (58 %) had genomic tumor profiles (A REMARK diagram is shown in Supplementary Figure S2). The baseline characteristics were comparable between the genomic cohort and the total study cohort (Supplementary Table S1), except for a higher frequency of surgical resection at diagnosis in the genomic cohort (89% vs. 81%, *P* = .03).

### Pattern of Progression and Prognostic Impact

**Figure 1.**
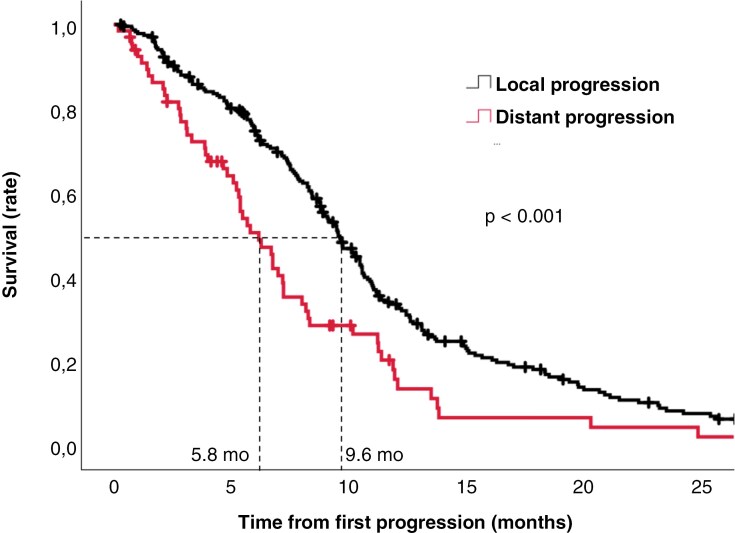
Kaplan–Meier curve showing the association between progression pattern and post-progression survival. The median post-progression survival for local progression and distant progression is shown.

The progression pattern was retrospectively reviewed and characterized in the 303 patients with radiologically confirmed progression. At the time of first progression, distant progression was found in 66 (22%) patients (Supplementary Figure S1). As shown in Table 1, the progression pattern was not significantly associated with time to tumor progression (median PFS: Local = 6.6 months vs. distant = 8.8 months, *P* = .10). On comparing the progression pattern with post-progression survival (Figure 1), patients with distant progression were significantly associated with a poor prognosis (Local = 9.6 months (95% CI: 8.6–10.5 months) versus distant = 5.8 months (95% CI: 4.6–7.1 months), *P* < .001). This poorer prognosis prevailed on adjusting for relapse surgery and second-line treatment (HR = 1.85, 95% CI: 1.35–2.52, *P* < .001).

### Distant Progression After First-Line Treatment

Of the patients with distant progression, 38 (58%) had a mixed progression pattern with both local and distant progression, and 29 patients (44%) had more than 1 new contrast-enhancing lesion (range 1–5). To determine if the distant tumor lesions were present and overlooked prior to oncological treatment, MRI scans from the time of diagnosis were systematically reviewed. Here, non-contrast-enhancing FLAIR signal changes at the site of the distant tumor were found in 3 patients (5 %). A median distance of 4 cm (range: 2.1–50 cm) was measured between the primary tumor and the most distantly positioned recurrent tumor. Of these patients, 2 (3%) experienced spread to the spinal cord in the form of a drop metastasis. A T2 FLAIR signal between the primary tumor and the recurrent tumor was recorded in 23 patients (35%).

Next, patients with distant progression were analyzed to evaluate if different progression patterns were associated with prognosis (post-progression survival): (1) mixed progression versus distant progression without local progression, (2) multiple new lesions versus 1 new lesion, (3) distance over 4 cm versus distance under 4 cm from the primary tumor, and (4) absence of a T2 FLAIR signal connection versus a T2 FLAIR signal connection to the primary tumor. In this analysis (shown in Supplementary Figure S3), mixed progression and multiple new lesions showed no significant impact on prognosis. Nevertheless, a longer distance (> 4 cm) from the primary tumor and the absence of a T2 FLAIR signal were associated with a significantly worse prognosis when compared to their respective counterparts (*P* < .02). Since longer distances (> 4 cm) between the tumors and lack of T2 FLAIR signal were significantly correlated (*P* < .001), the 2 factors were tested by multivariate analysis. This analysis demonstrated that distant lesions located more than 4 cm away from the primary tumor were significantly associated with a worse prognosis (HR: 2.62, 95% CI: 1.36–5.06, *P* = .004), while the absence of a T2 FLAIR signal showed no prognostic impact (*P* = .5).

Taken together, among the various radiographic features observed in distant progression, the most detrimental prognostic factor was the increased distance from the primary tumor.

### Distant Progression After Second-Line Treatment

Local progression was observed in 237 patients at the time of first progression (Supplementary Figure S1). Out of these, 191 patients received second-line treatment for their tumor progression and were subsequently followed with MRI. Among these patients, 41 (21%) later experienced distant progression of their tumor. Of those 41 patients, 19 (46%) had more than 1 new lesion, the median distance between the primary tumor and the most distantly positioned new lesion was 4.1 cm (range: 2.1–35 cm) and 13 patients (32%) were judged to have a T2 FLAIR signal between the primary lesion and the distant lesion. Mixed progression was not evaluated for these patients. A further 2 patients had a new lesion in the spinal cord (5%). When taken together with distant progression after first-line treatment, a total of 4 patients had a new lesion in the spinal cord. In summary, the pattern of progression after second-line treatment was comparable to the progression pattern after first-line therapy.

### Predictors of Distant Progression

Table 1 presents a comparison of the candidate baseline factors with progression patterns. The analysis revealed that patients with distant progression had less frequent p53 protein expression (*P* = .02) and tended to have less frequent subventricular zone involvement (*P* = .07) compared to patients with local progression. No other baseline factors were associated (*P* ≥ .2) with the incidence of distant progression.

Considering time to distant progression as the endpoint, we performed Cox regression analysis to identify baseline factors associated with time to distant progression. By univariate analysis, (shown in Supplementary Table S2), the following factors were found associated with shorter time to distant progression: younger age at baseline, male sex, non-resected tumor, extent of surgical resection, multicentric disease, non-temporal tumor location, parietal tumor location, unmethylated *MGMT*, and lack of p53 expression (*P* < .3). Of these, age at diagnosis (*P* = .19), non-resected tumor (*P* = .26), multicentric disease (*P* = .23), unmethylated *MGMT* (*P* = .002), and lack of p53 protein expression (*P* = .05) were chosen to be included in the multivariate model. The following baseline factors were not associated with time to distant progression: ECOG PS, subventricular zone involvement, and corticosteroid use.

The selected factors from the univariate analysis were subsequently analyzed together with known prognostic factors using multivariate analysis (Table 2). On adding p53 expression to the multivariate model, it was not significantly associated with distant progression (*P* = .11) and was therefore excluded. Of known independent prognostic factors, unmethylated *MGMT* was the only factor that was associated with a higher risk of developing distant progression (HR: 2.31, 95% CI: 1.31–4.07, *P* = .004). Age at diagnosis, biopsied (non-resected) tumors, and multicentric disease were not (*P* > .15) associated with a higher risk of distant progression. The c-index, performed as a measure of discrimination in the model, was 0.63. This result remained unchanged upon conducting a competing risk analysis. Therefore, the results and following analyses are shown without adjustment for competing risk.

**Table 2.** Multivariate Analysis of the Clinical Cohort Showing Association to Time to Distant Progression

	Distant Progression at the Time of First Progression	Distant Progression at the Time of First Progression or After Second-Line Treatment
Covariate	HR (95% CI) *P*-value	HR (95% CI) *P*-value
*MGMT* status, unmethylated vs. methylated	**2.31 (1.31–4.07)** **.004**	**2.54 (1.62–3.97)** **<.001**
Corticosteroid use^a^, yes vs. no	0.92 (0.54–1.58) .77	1.04 (0.69–1.58) .84
Age, per 10-year increase	0.97 (0.77–1.22) .79	0.95 (0.80–1.14) .61
Multicentric vs. single lesion	1.78 (.81–3.94) .15	**2.32 (1.28–4.20)** **.005**
ECOG performance status 1-2 vs. 0	0.99 (0.57–1.72) .97	0.83 (0.53–1.29) .40
Biopsy vs. resection	1.11 (0.54–2.28) .78	0.87 (0.48–1.58) .64
C-index	.63	.67

^a^Prednisolone dose > 10 mg/day at initiation of concomitant treatment.

Abbreviations: HR, Hazard ratio; CI, Confidence interval; *MGMT*, O6-methylguanine-DNA methyltransferase; C-index, concordance index. P values < 0.05 are considered significant and are made bold.

Lastly, we performed a multivariate analysis to evaluate if factors included in our multivariate model were associated with distant progression at the time of first progression or after second-line treatment. Out of 303 evaluable patients, 107 patients (35%) had distant progression at the time of first progression or after second-line treatment (Supplementary Figure S1). This multivariate analysis (Table 2) revealed that unmethylated *MGMT* (HR: 2.54, 95% CI: 1.62–3.97, *P* < .001) and multicentric disease (HR: 2.32, 95% CI: 1.28–4.20, *P* = .005) were independent predictors for shorter time to distant progression. The c-index for the model was 0.67.

Overall, unmethylated *MGMT* was significantly associated with a higher risk of distant progression at the time of first progression. When increasing the statistical power by modeling distant progression at any time-point, both unmethylated *MGMT* and multicentric disease were independent predictors for a shorter time to distant progression.

### Genomic Biomarkers Predictive of Distant Progression

**Figure 2.**
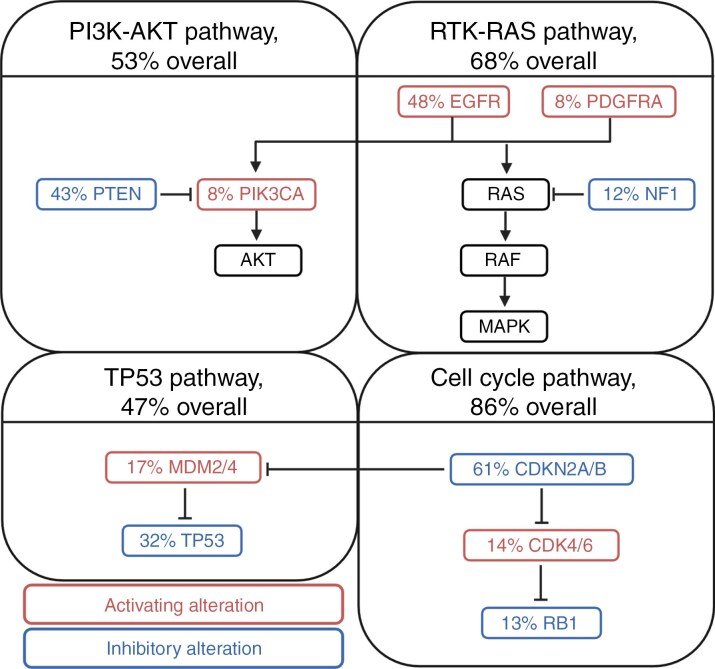
The 4 common glioblastoma signaling pathways and candidate genetic alterations belonging to them. The frequency of alterations identified in analyzed samples is also shown as a percentage.

The genomic cohort included 204 consenting patients. WES was performed on 125 patients (61%), WGS was performed on 75 patients (37%), and TSO500 was performed on 4 patients (2%). A total of 186 patients were evaluated for progression pattern and 35 patients (19%) were found to have distant progression (Supplementary Table S1). Eleven candidate genetic alterations and 4 altered signaling pathways were identified in over 5% of analyzed samples, as shown in Figure 2. Additionally, Supplementary Figure S4, an oncoprint, gives an overview of the genetic alterations and clinical characteristics of each patient. Neurofibromin 1 *(NF1)* gene alterations in patients experiencing either distant progression or local progression seem to coincide with the occurrence of a shorter PFS.

Subsequently, a univariate analysis was performed to identify associations between the genomic candidate factors and time to distant progression (Supplementary Table S3). Out of 11 candidate gene variants, loss-of-function alteration in the *NF1* gene (*P* = .001) and *PIK3CA* mutation (*P* = .19) were found to be associated with a higher risk of developing distant progression. Interestingly, mutations in the *RB1* gene were associated with a longer time to distant progression (*P* = .13). Alterations that were grouped according to the 4 altered GBM signaling pathways (cell cycle, TP53, PI3K, and RTK-RAS), revealed that gene alterations in the cell-cycle pathway (*P* = .03) and TP53 pathway (*P* = .07) had a protective effect and were associated with a longer time to distant progression.

On adjusting for unmethylated *MGMT* and multicentric disease in a multivariate analysis, only *NF1* alteration was associated with a shorter time to distant progression (HR: 3.48, 95% CI: 1.48–8.21, *P* = .004) (Table 3). The c-index for the model was 0.70. *PIK3CA* mutation (*P* = .28), mutations in the *RB1* gene (*P* = .32), and alterations in the cell cycle pathway (*P* = .08) and TP53 pathway (*P* = .09) were not associated with distant progression.

**Table 3.** Multivariate Analysis of the Genomic Cohort Showing Association to Time to Distant Progression

Covariate	HR (95% CI) *P*-value
*NF1* alteration vs. *NF1* wildtype	**3.48 (1.48–8.21)** **.004**
*MGMT* status, unmethylated vs. methylated	**3.10 (1.43–6.74)** **.004**
Multicentric vs. single lesion	1.24 (0.37–4.14) .72
C-index	.70

Abbreviations: HR, Hazard ratio; CI, Confidence interval; *MGMT,* O6-methylguanine-DNA methyltransferase; *NF1,* Neurofibromin 1; C-index, concordance index P values < 0.05 are considered significant and are made bold.

### Baseline Characteristics and Survival in *NF1*-Altered Tumors

Pathogenic or likely pathogenic *NF1* gene alterations were identified in 23 patients, with 7 of these patients (30%) developing distant progression. An overview of the locus of alterations, type of mutations, and pattern of progression is shown in Supplementary Table S4. Most of the mutations were frameshift or nonsense mutations leading to a truncated or nonfunctional neurofibromin protein. No mutation hotspots were identified. Additionally, no germline mutations in the *NF1* gene were detected, though this was not tested systematically. Chi-square analysis showed no significant associations between clinical and radiological baseline factors and *NF1* gene alterations (Supplementary Table S5). However, there was a significant association between *NF1* alterations and the absence of epidermal growth factor receptor *(EGFR)* gene amplification (*P* < .001) or phosphatidylinositol-4,5-bisphosphate 3-kinase catalytic subunit alpha (*PIK3CA*) gene mutations (*P* = .008).

**Figure 3.**
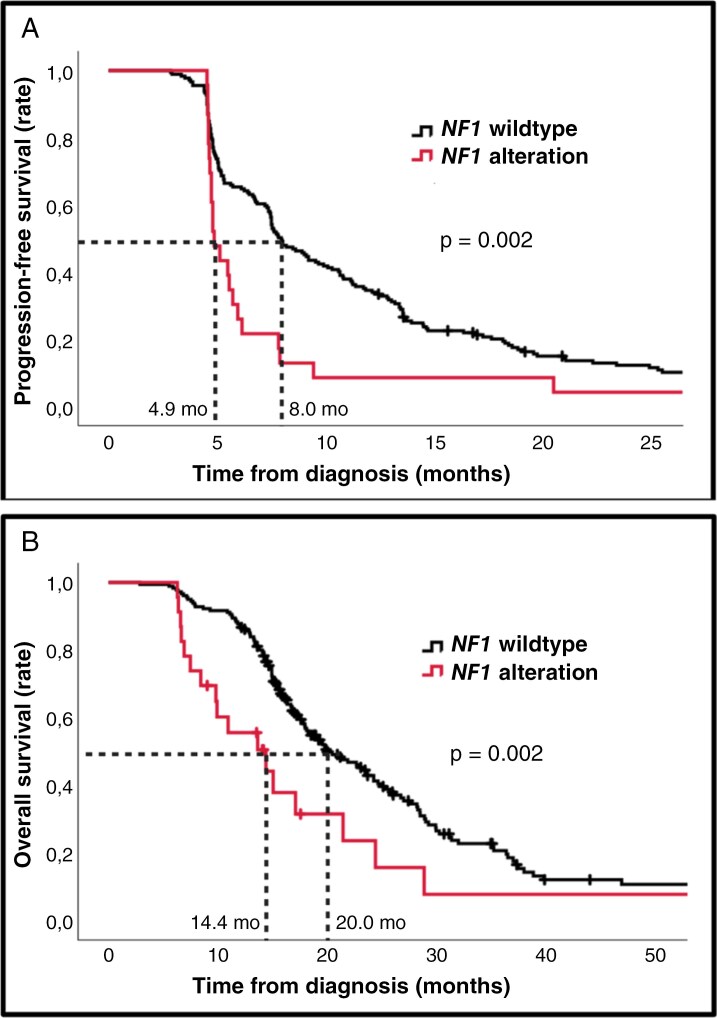
(A) Kaplan–Meier curve showing the association between progression-free survival and *NF1* gene status. (B) Kaplan–Meier curve showing the association between overall survival and *NF1* gene status. The median progression-free survival and overall survival for both groups are shown. In multivariate analysis adjusted for known prognostic factors such as, age, ECOG performance status, multicentric disease, corticosteroid use, resection versus biopsy and, *MGMT* status, loss-of-function *NF1* alteration remained independently associated with a poor OS (hazard ratio: 2.0, 95% confidence interval): 1.2–3.3, *P* = .008).

As shown in Figure 3, patients with *NF1* alterations, compared to *NF1* wildtype, had a significantly shorter PFS (4.9 months vs. 8.0 months, *P* = .002) and OS (14.4 months vs. 20.0 months, *P* = .002). Loss-of-function *NF1* alteration remained independently associated with a poor OS (HR: 2.0, 95% CI: 1.2–3.3, *P* = .008) after adjusting for known prognostic factors such as age, ECOG PS, multicentric disease, corticosteroid use, resection vs biopsy, and *MGMT* status in a multivariate analysis.

## Discussion

In this study involving newly diagnosed GBM *IDH*wt patients receiving standard therapy, we analyzed clinical, radiological, and genetic factors and their associations with distant tumor progression. Three independent predictors, unmethylated *MGMT,* multicentric disease, and *NF1* gene alterations, were found to be correlated with a shorter time to distant progression.

In the context of this study, distant progression was defined as a new contrast-enhancing lesion more than 2 cm from the primary tumor or surgical cavity. Although this definition is the most used, the concept of distant progression patterns has varied across previous studies. Some researchers have employed different distance metrics, while others have defined progression patterns based on radiation volumes according to the 95% isodose line.^20^ This dissent makes it challenging to compare results across studies. Nevertheless, the incidence of distant progression observed in this study (22%) was comparable to findings from other studies, where distant progression varied from 21 to 28 %.^3,5,8,10,21,22^ Thus, we suggest that the progression pattern observed in our study cohort is representative of the general population of GBM patients undergoing standard therapy.

In support of previous findings, we found that patients experiencing distant progression had significantly poor post-progression survival compared to patients with local progression.^6,8,9,23^ This association remained significant when adjusting for salvage surgery and second-line treatment, suggesting that MRI-visible tumors spreading far from the primary site exhibit an advanced and more aggressive migratory phenotype.

In our analysis of patients with distant progression, signal changes at distant tumor sites were rarely (5%) found at the time of diagnosis, suggesting that distant progression most often reflected tumor cell migration due to treatment failure. Nevertheless, the possibility of preexisting microscopic clones growing at the distant tumor site, as suggested in a previous study, cannot be excluded based on our analysis.^24^

In distantly progressing tumors, we observed substantial heterogeneity in radiographic features, including 4 cases of drop metastasis, demonstrating that not all tumors progress by direct brain infiltration. However, in other cases, we were unable to differentiate between mechanisms of spread. Interestingly, the prognosis for patients with either multiple new lesions or combined local and distant progression was comparable to that of patients with a solitary distant tumor lesion. However, it is important to highlight that half of the patients with distant progression had tumor lesions located more than 4 cm from the primary tumor or surgical cavity, and this subgroup demonstrated significantly worse post-progression survival. Taken together, these findings suggest that the distance from the primary tumor site to the most distantly progressing lesion is the most significant prognostic radiographic feature in patients with distant progression.

On screening multiple factors for association with time to distant progression, we found that unmethylated promoter of the *MGMT* gene was significantly associated with a shorter time to distant progression. This finding contradicts previous studies that have linked methylated *MGMT* with a higher incidence of distant progression.^8,25,26^ One possible explanation is that MGMT promoter methylation is associated with improved survival and longer follow-up, potentially leading to more frequent observations of distant progression.^8,27^ It could also be a result of differences in study cohorts, sample size, and the statistical methods used, as these studies did not consider time to distant progression. Corroborating our results, data from an earlier cohort also showed a significantly shorter time to distant progression in patients with unmethylated *MGMT*.^23^ Collectively, our findings suggest that reduced chemosensitivity in patients with *MGMT* unmethylated GBMs not only leads to impaired local tumor control^28^ but also impaired distant tumor control.

Moreover, patients presenting with multicentric disease at the time of diagnosis exhibited a borderline significant association with a higher risk of developing distant progression at the time of first progression. When including those who experienced distant progression after second-line treatment, this association became significant. To the best of our knowledge, we are the first to show that multicentric tumors are more likely to progress distantly from the primary tumor lesions.^29–31^ Although this progression pattern is a result of multiple factors, a likely explanatory factor could be the multicentric tumor’s intrinsic ability to progress by new isolated contrast-enhancing tumors.^29^

In our study, pathogenic or likely pathogenic *NF1* loss-of-function alteration was independently associated with a higher risk of distant progression. Furthermore, and in line with recent studies, the *NF1* alteration was an independent prognostic factor associated with a poor PFS and OS.^32–34^ The NF1 protein is a negative regulator of the RAS-MAPK pathway. Therefore, loss-of-function alterations in the *NF1* gene lead to hyperactivation of RAS-MAPK signaling, potentiating cell proliferation, and migration.^35,36^ This could explain the association between *NF1* alteration and the higher risk of distant progression and poor prognosis.

In GBM, the RAS-RAF-MAPK pathway is most frequently affected by activating alterations to the membrane receptors, such as EGFR, and more rarely by genetic alterations of downstream effectors such as NF1 and BRAF.^37^ Interestingly, we found that tumors harboring an *NF1* alteration were not associated with co-mutations in the *EGFR* gene or the *PIK3CA* gene. Consequently, in patients with *NF1*-altered GBM, drugs targeting downstream proteins may demonstrate enhanced efficacy. To date, effective drugs targeting downstream molecules involved in this pathway are limited to BRAF-inhibitors in *BRAF*-mutated gliomas.^38^ However, preclinical and clinical studies have shown efficacy of MEK-inhibitors in *NF1*-altered GBMs but not in *NF1* wildtype tumors.^39–44^ Accordingly, *NF1* alteration may serve as a predictive factor for therapies targeting the RAS-RAF-MAPK pathway, such as MEK-inhibitors. We hypothesize that in newly diagnosed *NF1*-altered GBM patients, inhibition of RAS-RAF-MAPK-induced migration, together with local treatment, could improve tumor control and survival.^45^

A previous study by Jiang et al. found that tumors in contact with the subventricular zone were associated with an increased risk of distant progression.^8^ In our study, Cox regression analysis found no significant association between subventricular zone involvement and a shorter time to distant progression (*P* = .44). Rather, a Chi-square test indicated that tumors in contact with the subventricular zone less frequently progressed distantly, though this association was not statistically significant (*P* = .07). Overall, these findings are consistent with previous studies, which also find no clear link between subventricular zone involvement and distant progression.^5,9^

This study suffers from some limitations. Firstly, the study is limited by the single-center study design and the sample size. Secondly, the risk of death or clinical progression prior to radiological evaluation poses a competing risk. Nevertheless, we performed a competing risk adjustment analysis and found that competing risk did not influence the study results. Thirdly, we cannot exclude the occurrence of pseudo-progression in our study. However, we have minimized the influence of pseudo-progression on our results by conducting a follow-up MRI scan 3 months after completing radiation therapy. For patients with suspected progression, imaging findings and clinical status (including corticosteroid use) were reviewed at multidisciplinary conferences to distinguish true progression from pseudo-progression, and in select cases, a confirmatory MRI scan was performed. Additionally, the distantly progressing lesions identified by using the “2 cm distance” definition are expected to be located outside the radiation treatment field, further reducing the likelihood of pseudo-progression. This is supported by the poor survival outcomes that are observed in patients with distant progression. Finally, this study was based on genomic reports rather than advanced bioinformatics analysis, which could potentially better identify key drivers of migratory GBM growth.

## Conclusion

Distant progression after standard therapy is an aggressive progression pattern associated with a poor prognosis. This progression pattern is influenced by multiple factors, of which we identified unmethylated *MGMT*, multicentric tumors at the time of diagnosis, and the presence of *NF1* gene alteration. We hypothesize that *NF1* alteration serves as a predictive biomarker for MEK-inhibition targeting migratory growth in newly diagnosed glioblastoma patients.

## Supplementary Material

vdaf092_suppl_Supplementary_Figure_S1

vdaf092_suppl_Supplementary_Figure_S2

vdaf092_suppl_Supplementary_Figure_S3

vdaf092_suppl_Supplementary_Figure_S4

vdaf092_suppl_Supplementary_Materials

## Data Availability

De-identified clinical and molecular data used for figures and analyses can be provided by the corresponding author upon request.
