# Supplementary figures and images for: Identifying targetable alterations predictive of distant progression in glioblastoma patients undergoing standard therapy
Source: Neurooncol Adv. 2025 May 7;7(1):vdaf092. doi: 10.1093/noajnl/vdaf092 (PMC12202037; doi:10.1093/noajnl/vdaf092)

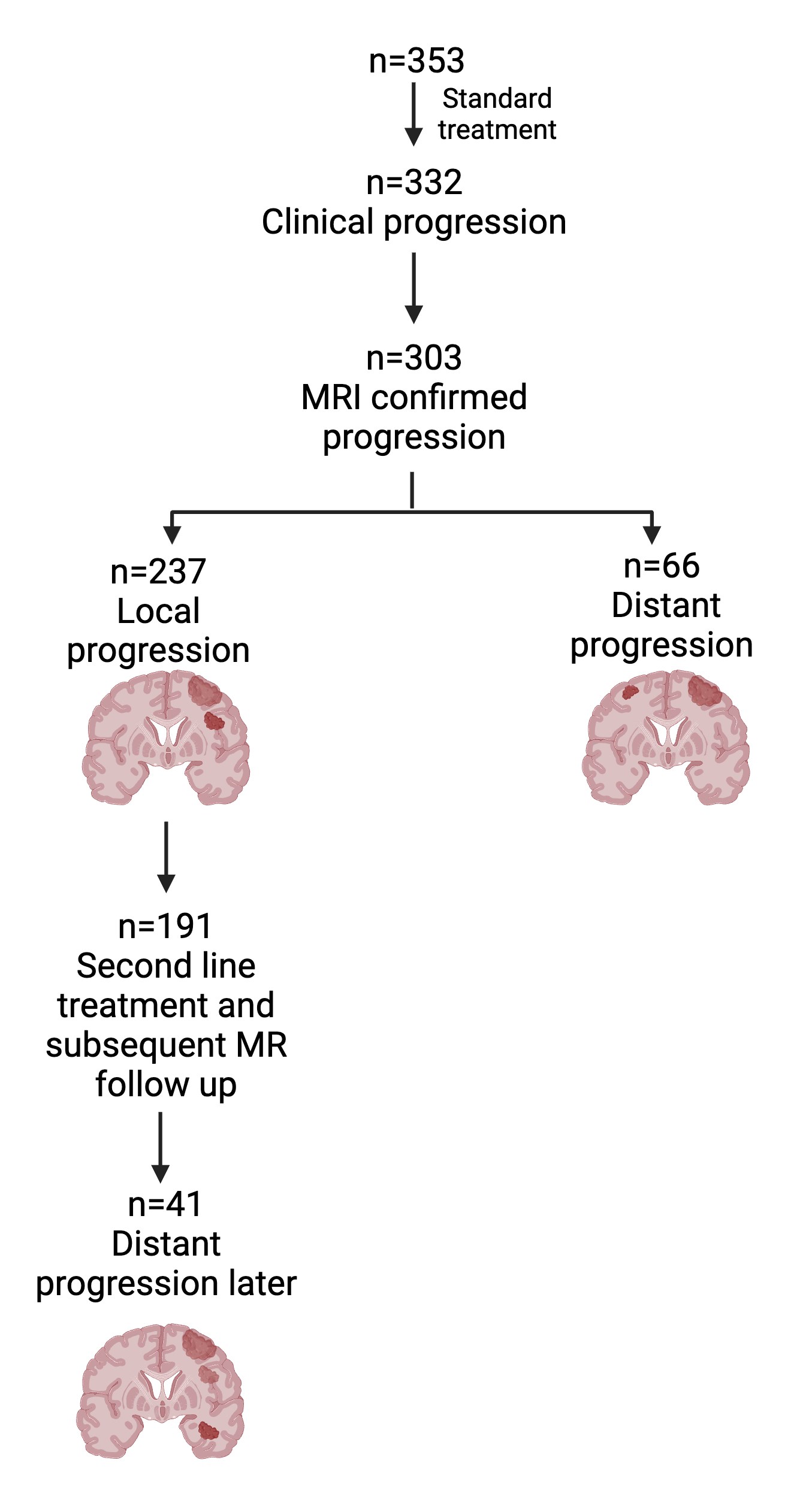

Supplement: vdaf092_suppl_Supplementary_Figure_S1 [file vdaf092_suppl_supplementary_figure_s1.jpeg]

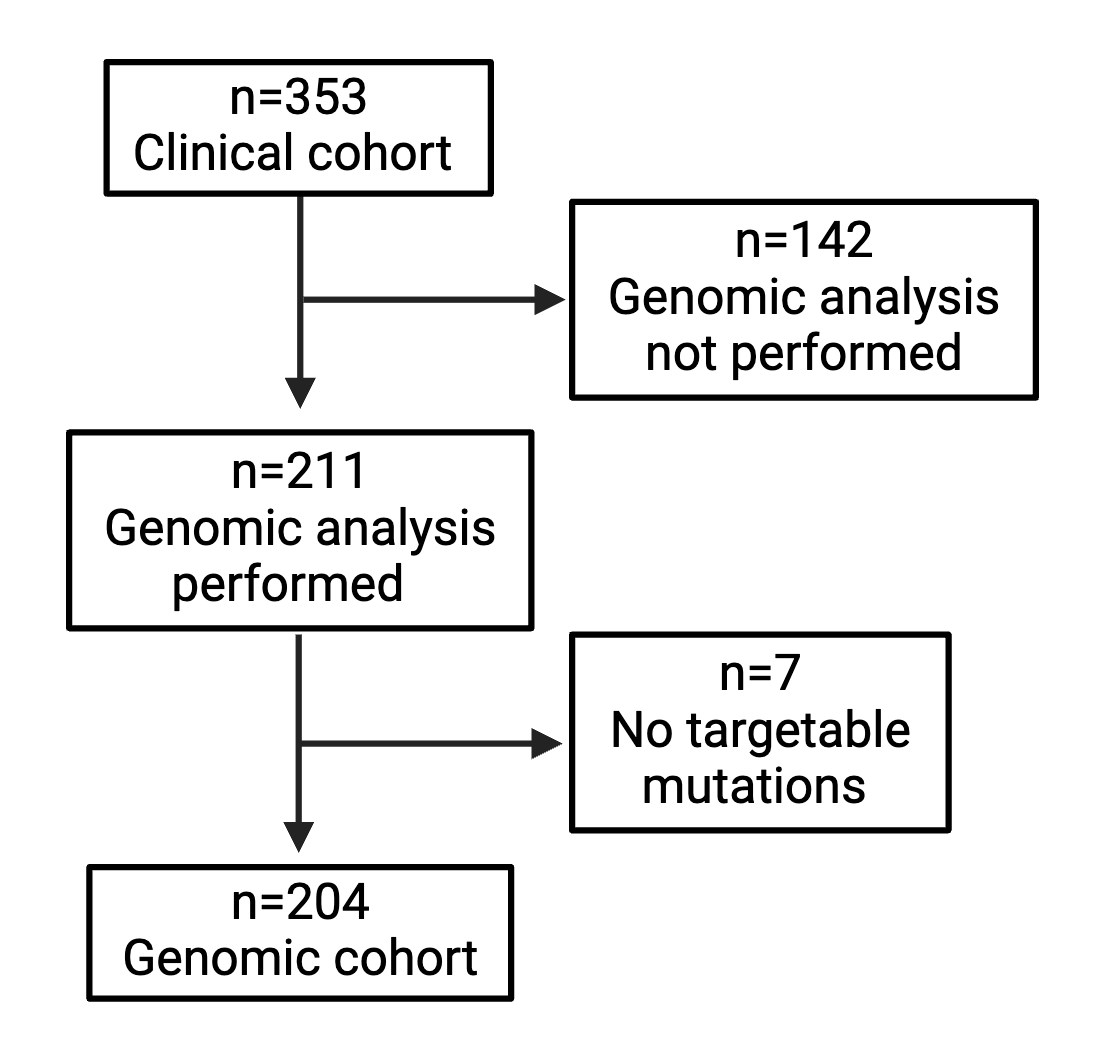

Supplement: vdaf092_suppl_Supplementary_Figure_S2 [file vdaf092_suppl_supplementary_figure_s2.jpeg]

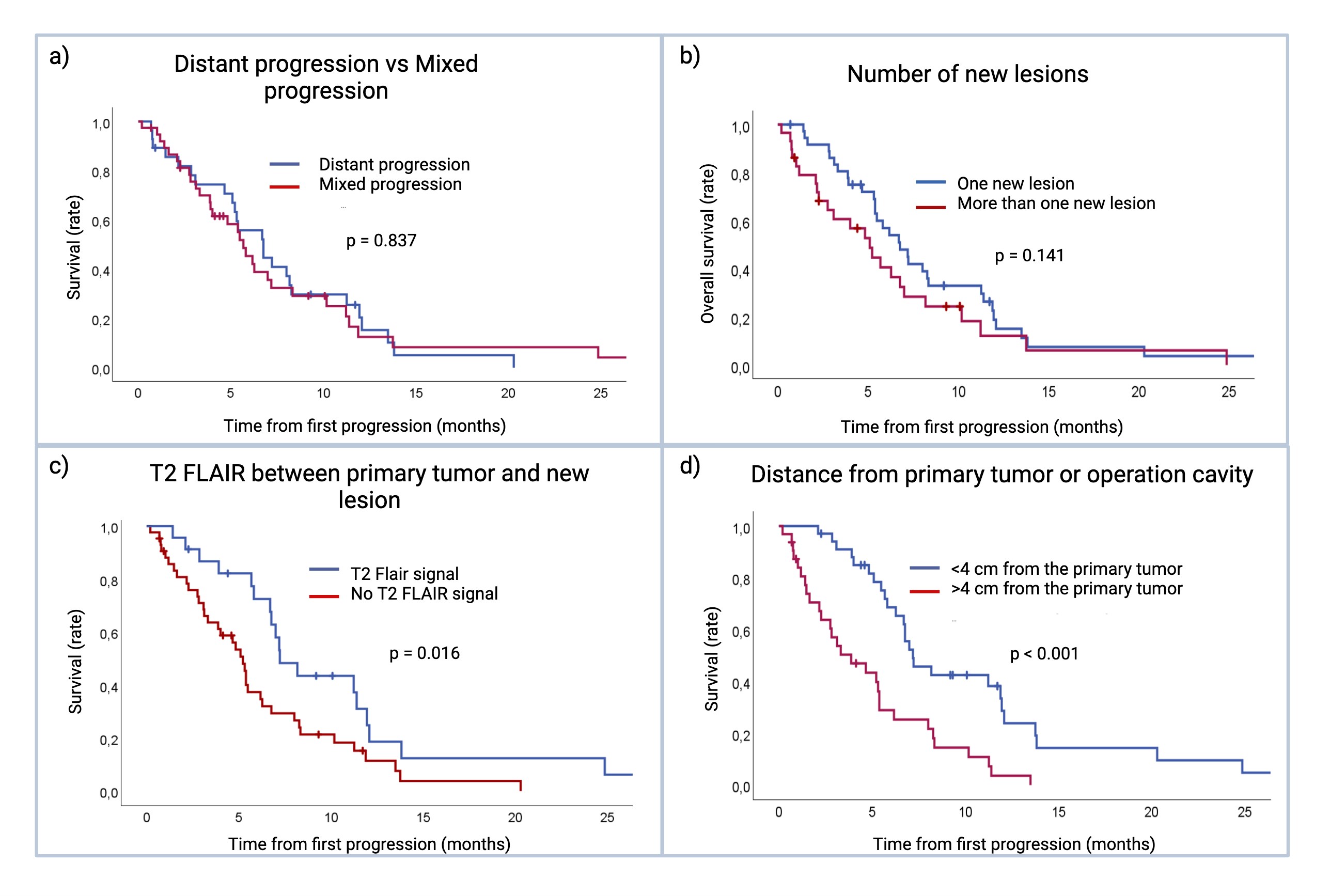

Supplement: vdaf092_suppl_Supplementary_Figure_S3 [file vdaf092_suppl_supplementary_figure_s3.jpeg]

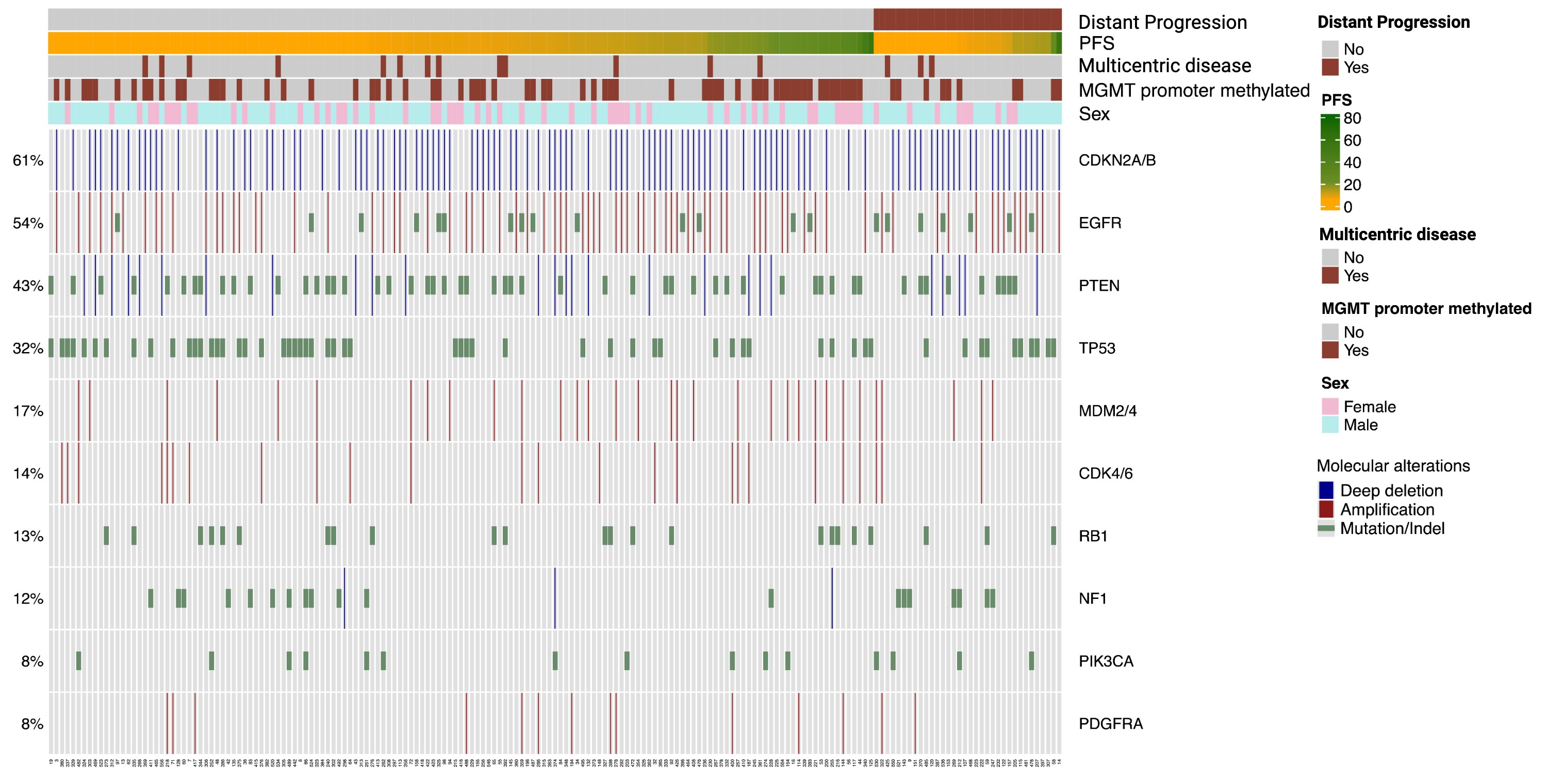

Supplement: vdaf092_suppl_Supplementary_Figure_S4 [file vdaf092_suppl_supplementary_figure_s4.jpeg]
