# Supplementary material for: Identifying targetable alterations predictive of distant progression in glioblastoma patients undergoing standard therapy
Source: Neurooncol Adv. 2025 May 7;7(1):vdaf092. doi: 10.1093/noajnl/vdaf092 (PMC12202037; doi:10.1093/noajnl/vdaf092)
Supplement: vdaf092_suppl_Supplementary_Materials [file vdaf092_suppl_supplementary_materials.docx]

| **Supplementary Table S1: Comparison of patient characteristics from clinical cohort and genomic cohort** | | | |
| --- | --- | --- | --- |
|  | **Total**  **n = 353** | **Genomic cohort**  **n = 204** | **p-value** |
| Median age, years (range) | 59 (17 – 77) | 58 (18-77) | 0.48 |
| Gender, *n* (%) |  |  | 0.39 |
| Female | 134 (38) | 70 (34) |  |
| Male | 219 (62) | 134 (66) |  |
| ECOG performance status, *n* (%) |  |  | 0.31 |
| 0 | 208 (60) | 133 (66) |  |
| 1 | 124 (35) | 60 (30) |  |
| 2 | 17 (5) | 8 (4) |  |
| Missing | 4 | 3 |  |
| Subventricular zone involvement, *n* (%) |  |  | 0.70 |
| yes | 153 (43) | 85 (42) |  |
| no | 200 (57) | 119 (58) |  |
| Multicentric disease, *n* (%) |  |  | 0.11 |
| Yes | 45 (13) | 17 (8) |  |
| No | 307 (87) | 187 (92) |  |
| Missing | 1 | 0 |  |
| Tumor localization, *n* (%) |  |  | 0.82 |
| Frontal lobe | 99 (28) | 57 (28) |  |
| Temporal lobe | 86 (24) | 55 (27) |  |
| Parietal lobe | 40 (11) | 24 (12) |  |
| Occipital lobe | 9 (3) | 5 (2) |  |
| Overlapping lobes | 95 (27) | 55 (27) |  |
| Not defined | 24 (7) | 8 (4) |  |
| Corticosteroid use^a^, *n* (%) |  |  | 0.17 |
| Yes | 161 (46) | 82 (40) |  |
| No | 186 (54) | 121 (60) |  |
| Missing | 6 | 1 |  |
| Resection, *n* (%) |  |  | **0.03** |
| Biopsy | 65 (19) | 23 (11) |  |
| Resection | 286 (81) | 180 (89) |  |
| Missing | 2 | 1 |  |
| Extent of surgical resection,  *n* (%) |  |  | 0.73 |
| Gross total resection | 98 (39) | 67 (40) |  |
| Near-total resection | 88 (35) | 61 (37) |  |
| Subtotal resection | 68 (27) | 39 (23) |  |
| Missing | 99 | 37 |  |
| *MGMT* status, *n* (%) |  |  | 0.72 |
| Methylated | 148 (42) | 91 (45) |  |
| Unmethylated | 205 (58) | 113 (55) |  |
| p53, *n* (%) |  |  | 0.82 |
| Positive | 215 (68) | 129 (69) |  |
| Negative | 103 (32) | 59 (31) |  |
| Missing | 35 | 16 |  |
| Recurrence pattern,  *n* (%) |  |  | 0.43 |
| Local | 237 (78) | 151 (81) |  |
| Distant | 66 (22) | 35 (19) |  |
| Missing | 50 | 18 |  |
| Median PFS 95% CI | 7.5 (6.8 – 8.2) | 7.5 (7.0 – 8.0) | 0.62 |
| Median OS 95% CI | 17.2 (15.8 – 18.7) | 19.7 (17.0 – 22.5) | 0.08 |
| Resection at recurrence,  *n* (%) |  |  | **0.01** |
| Yes | 115 (35) | 88 (46) |  |
| No | 215 (65) | 104 (54) |  |
| Missing | 23 | 12 |  |
| Second line treatment,  *n* (%) |  |  | **0.02** |
| Yes | 240 (74) | 158 (84) |  |
| No | 84 (26) | 29 (16) |  |
| Missing | 29 | 17 |  |
| ^a^Prednisolone dose > 10 mg/day at initiation of concomitant treatment.  Abbreviations*:*  *MGMT:* O6-methylguanine-DNA methyltransferase;  PFS: progression free survival;  OS: overall survival | | | |

| **Supplementary Table S2. Clinical cohort - Univariate analysis modelling time to distant recurrence** | | |
| --- | --- | --- |
| **Covariate** | **HR**  **(95% CI)** | ***p*-value** |
| Age, per 10-year increase | 0.87 (0.71 – 1.07) | **0.19** |
| Gender (female *vs.* male) | 0.74 (0.44 – 1.25) | **0.26** |
| ECOG performance status 1-2 *vs*. 0 | 0.87 (0.52 – 1.44) | 0.58 |
| Resection (reference = biopsy) | 0.69 (0.36 – 1.32) | **0.26** |
| Extent of surgical resection (reference = subtotal resection) |  |  |
| Gross total resection | 0.87 (0.51 – 1.50) | 0.62 |
| Near-total resection | 0.43 (0.21 – 0.88) | **0.02** |
| Multicentric *vs.* single lesion | 1.58 (0.75 – 3.34) | **0.23** |
| Subventricular zone yes *vs.* no | 0.81 (0.48 – 1.37) | 0.44 |
| Frontal location *vs.* non-frontal location | 0.97 (0.57 – 1.64) | 0.91 |
| Temporal location *vs.* non-temporal location | 0.44 (0.20 – 0-96) | **0.04** |
| Parietal location *vs.* non-parietal location | 1.45 (0.76 – 2.77) | **0.26** |
| Occipital location *vs.* non-occipital location | 1.17 (0.29 – 4.81) | 0.82 |
| Overlapping lobes vs non-overlapping lobes | 1.30 (0.78 – 2.17) | 0.32 |
| Corticosteroid use^a^ (yes *vs.* no) | 1.18 (0.72 – 1.92) | 0.52 |
| *MGMT* unmethylated *vs.* methylated | 2.23 (1.33 – 3.74) | **0.002** |
| p53 expression yes vs. no | 0.61 (0.37 – 0.99) | **0.05** |
| ^a^Prednisolone dose > 10 mg/day at initiation of concomitant treatment.  *Abbreviations:*  HR: Hazard ratio  CI: Confidence interval  *MGMT:* O6-methylguanine-DNA methyltransferase; | | |

| **Supplementary Table S3: Univariate analysis of genomic cohort showing association to time to distant progression** | |
| --- | --- |
| **Genetic variants, n (%)** | **HR (95%CI)**  **p-value** |
| *CDKN2A/B*-del, 126 (61%) | 1.24 (0.63-2.45)  0.54 |
| *EGFR-*amp, 97 (48%) | 1.20 (0.62-2.33)  0.59 |
| *TP53*-mut, 64 (32%) | 0.91 (0.43-1.90)  0.80 |
| *PTEN-*alteration, 87 (43%) | 1.15 (0.58-2.26)  0.69 |
| *MDM2/4*-amp, 33 (17%) | 0.81 (0.31-2.08)  0.66 |
| *EGFR*-mut, 31 (15%) | 1.05 (0.44-2.53)  0.91 |
| *CDK4/6-*amp, 26 (14%) | 0.80 (0.24-2.61)  0.71 |
| *RB1*-mut, 28 (13%) | **0.39 (0.12-1.33)**  **0.13** |
| *NF1*-alteration, 23 (12%) | **3.96 (1.70-9.20)**  **0.001** |
| *PIK3CA*-mut, 16 (8%) | **2.00 (0.70-5.68)**  **0.19** |
| *PDGFRA-*fusion/amp, 18 (8%) | 0.86 (0.21-3.57)  0.83 |
| Altered signaling pathways (yes vs no) n (%) |  |
| Cell cycle pathway, 176 (86%) | **0.45 (0.22 – 0.91) 0.03** |
| TP53 pathway, 97 (47%) | **0.57 (0.32-1.04)**  **0.07** |
| PI3K pathway, 108 (53%) | 0.89 (0.51 – 1.53)  0.67 |
| RTK-RAS pathway, 139 (68%) | 1.00 (0.61 – 1.64)  0.99 |
| Genetic alterations were either (1) mutations, defined as pathogenic or likely pathogenic single-nucleotide variants, insertions/deletions and fusions (2) amplifications (3) biallelic deletions  The signaling pathways were considered altered when when one or more genes belonging to the pathway was altered.  Abbreviations:  HR: Hazard ratio  CI: Confidence interval  Mut: mutations defined as single nucleotide variants and insertions/deletions  Amp: Amplification  Del: Biallelic deletion | |

| Supplementary Table S4: Overview of locus of alterations, type of mutations and pattern of progression in patients with *NF1* altered tumors | | | | | |
| --- | --- | --- | --- | --- | --- |
| ID | *NF1* locus 1 | *NF1* locus 2 | *NF1* locus 3 | Type of mutation | Pattern of progression |
| 1 | c.1888delG, p.V630* |  |  | Nonsense mutation | Distant |
| 2 | c.1746C>A, p .C582 |  |  | Nonsense mutation | Distant |
| 3 | c.7908-2A>G | c.6683T>C, p.L2249P (VUS) |  | Splice-site mutation and missense mutation | Distant |
| 4 | c.4855G>T, p.E1619* |  |  | Stop-gain variant | Distant |
| 5 | c.6756+1G>A | c.7316C>A, p.S2439* |  | Splice site mutation and nonsense mutation | Distant |
| 6 | c.5565_5567delTCT, p.L1856del |  |  | 3-bp-in-frame-deletion | Distant |
| 7 | c.3779_3742del, p.F1247fs*18 |  |  | Frameshift mutation | Distant |
| 8 | c.4991G>A, p.W1664* |  |  | Missense mutation | Local |
| 9 | Biallelic deletion: NF1 |  |  | Biallelic deletion | Local |
| 10 | c.3916C>T, p.R1306* | c.1756_1759delACTA, p.T586fs*18 |  | Nonsense mutation and frameshift mutation | Local |
| 11 | c.889-1G>A |  |  | Splice-site loss | Local |
| 12 | c.3861_3862delCT, p.F1287fs*26 |  |  | Frameshift mutation | Local |
| 13 | c.3479delG, p.G1160fs*6 | c.3739_3742delTTTG, p.F1247fs* |  | 2 frameshift mutations | Local |
| 14 | Biallelic deletion: NF1 |  |  | Biallelic deletion | Local |
| 15 | c.5609G>A, p.R1870Q | c.5268+3_5268+6del |  | Missense mutation and frameshift mutation | Local |
| 16 | c.6789_6792delTTAC, p.Y2264fs* |  |  | Frameshift mutation | Local |
| 17 | c.3822_3823delCT, p.F1275fs*8 | c.7342G>T, p.E2448 |  | Frameshift mutation and nonsense mutation | Local |
| 18 | c.479+2T>G (splice site loss) | c.4108C>T, p.Q1370* |  | Splice site loss and frameshift mutation | Local |
| 19 | c.7996_7997delAG, p.S2666fs*5 |  |  | Frameshift mutation | Local |
| 20 | c.1318C>T, p.R440* | c.4157delA, p.K1386fs*20 | c.7348C>T, p.R2450* | 2 stop-gain variants and 1 frameshift mutation | Local |
| 21 | Biallelic deletion: NF1 |  |  | Biallelic deletion | Local |
| 22 | c.5839C>T, p.R1947* | c.4860dupT, p.V1621fs*10 |  | Nonsense mutation and frameshift mutation | Local |
| 23 | c.5902C>T, p.R1968* |  |  | Nonsense mutation | Local |
| *Abbreviations:*  *NF1:* Neurofibromin 1  ID: Identity number | | | | | |

| Supplementary Table S5: Patient characteristics: *NF1-*wildtype vs *NF1-*altered tumors | | | |
| --- | --- | --- | --- |
|  | *NF1-*wildtype  n = 181 | *NF1-*altered  n = 23 | p-value |
| Median age, years (range) | 58 (18-77) | 60 (33 – 73) | 0.57 |
| Gender, *n* (%) |  |  | 0.68 |
| Female | 63 (35) | 7 (30) |  |
| Male | 118 (65) | 16 (70) |  |
| ECOG performance status, *n* (%) |  |  | 0.57 |
| 0 | 120 (67) | 13 (57) |  |
| 1 | 51 (29) | 9 (39) |  |
| 2 | 7 (4) | 1(4) |  |
| Missing | 3 | 0 |  |
| Subventricular zone involvement, *n* (%) |  |  | 0.13 |
| yes | 72 (40) | 13 (57) |  |
| no | 109 (60) | 10 (43) |  |
| Multicentric disease, *n* (%) |  |  | 0.13 |
| Yes | 17 (9) | 0 (0) |  |
| No | 164 (91) | 23 (100) |  |
| Tumor localization, *n* (%) |  |  | 0.42 |
| Frontal lobe | 52 (29) | 5 (22) |  |
| Temporal lobe | 50 (28) | 5 (22) |  |
| Parietal lobe | 19 (10) | 5 (22) |  |
| Occipital lobe | 4 (2) | 1 (4) |  |
| Overlapping lobes | 50 (28) | 5 (22) |  |
| Not defined | 6 (3) | 2 (8) |  |
| Corticosteroid use^a^, *n* (%) |  |  | 0.90 |
| Yes | 73 (41) | 9(39) |  |
| No | 107 (59) | 14 (61) |  |
| Missing | 1 | 0 |  |
| Resection, *n* (%) |  |  | 0.78 |
| Biopsy | 20 (11) | 3 (13) |  |
| Resection | 160 (89) | 20 (87) |  |
| Missing | 1 | 0 |  |
| Extent of surgical resection, *n* (%) |  |  | 0.92 |
| Gross total resection | 59 (40) | 8 (45) |  |
| Near-total resection | 55 (37) | 6 (33) |  |
| Subtotal resection | 35 (23) | 4(22) |  |
| Missing | 32 | 5 |  |
| *MGMT* status, *n* (%) |  |  | 0.31 |
| Methylated | 83 (46) | 8 (35) |  |
| Unmethylated | 98 (54) | 15 (65) |  |
| p53, *n* (%) |  |  | 0.06 |
| Positive | 110 (66) | 19 (86) |  |
| Negative | 56 (34) | 3 (14) |  |
| Missing | 15 | 1 |  |
| Recurrence pattern, *n* (%) |  |  | 0.1 |
| Local | 136 (83) | 15 (68) |  |
| Distant | 28 (17) | 7 (32) |  |
| Missing | 17 | 1 |  |
| Median PFS 95% CI | 8.0 (6.9 – 9.0) | 4.9 (4.4 – 5.3) | 0.002 |
| Median OS 95% CI | 20 (16.5 – 23.4) | 14.4 (7.8 – 20.9) | 0.002 |
| Resection at recurrence, *n* (%) |  |  | 0.49 |
| Yes | 79 (47) | 9 (39) |  |
| No | 90 (53) | 14 (61) |  |
| Missing | 12 | 0 |  |
| Second line treatment, *n* (%) |  |  | 0.38 |
| Yes | 140 (85) | 18 (78) |  |
| No | 24 (15) | 5 (22) |  |
| Missing | 17 | 0 |  |
| *CDKN2A/B*-del, *n* (%) |  |  | 0.93 |
| Yes | 112 (62) | 14 (61) |  |
| No | 69 (38) | 9(39) |  |
| *EGFR*-amp, *n* (%) |  |  | <0.001 |
| Yes | 95 (53) | 2 (9) |  |
| No | 83 (47) | 21 (91) |  |
| Missing | 3 | 0 |  |
| *TP53*-mut, *n* (%) |  |  | 0.71 |
| Yes | 56 (31) | 8 (35) |  |
| No | 125 (69) | 15 (65) |  |
| *PTEN*-alteration, *n* (%) |  |  | 0.93 |
| Yes | 77 (42) | 10 (44) |  |
| No | 104 (58) | 13 (56) |  |
| *MDM2/4*-amp, *n* (%) |  |  | 0.67 |
| Yes | 30 (17) | 3 (13) |  |
| No | 151 (83) | 20 (87) |  |
| *EGFR*-mut, *n* (%) |  |  | 0.12 |
| Yes | 30 (17) | 1 (4) |  |
| No | 151 (83) | 22 (96) |  |
| *CDK4/6*-amp, *n* (%) |  |  | 0.05 |
| Yes | 26 (14) | 0 (0) |  |
| No | 155 (86) | 23 (100) |  |
| *RB1*-mut, *n* (%) |  |  | 0.92 |
| Yes | 25 (14) | 3(13) |  |
| No | 156 (86) | 20 (87) |  |
| *PIK3CA*-mut, *n* (%) |  |  | 0.008 |
| Yes | 11 (6) | 5(22) |  |
| No | 170 (94) | 18 (78) |  |
| *PDGFRA*-fusion/amp, *n* (%) |  |  | 0.11 |
| Yes | 18 (10) | 0(0) |  |
| No | 163 (90) | 23 (100) |  |
| ^a^Prednisolone dose > 10 mg/day at initiation of concomitant treatment.  *Abbreviations:*  *NF1:* Neurofibromin 1  *MGMT*: O6-methylguanine-DNA methyltransferase;  PFS: progression free survival;  OS: overall survival  Mut: mutations defined as single nucleotide variants and insertions/deletions  Amp: Amplification  Del: Biallelic deletion | | | |

**FIGURE LEGENDS**

**Supplementary Figure S1:** Flowchart showing the chronological order of events in which patients have experienced distant progression

**Supplementary Figure S2:** REMARK diagram showing the selection of participants in the genomic cohort

**Supplementary Figure S3:** Kaplan Meier curves showing association between subtypes of distant progression and post-progression survival. a) Mixed progression is the simultaneous occurrence of distant progression and local progression. The kaplan meier curve explores the differences in prognosis between those patients experiencing isolated distant progression and experiencing mixed progression. b) this diagram explores differences in prognosis in patients experiencing one new lesion vs patients experiencing multiple new lesions. c) This diagram shows the differences in post-progression survival between patients that have a T2 FLAIR signal connection between their distantly progressing lesion and primary tumor and those lacking a T2 FLAIR signal. d) This diagram shows differences in prognosis between patients experiencing a new lesion that lies over 4 cm away from the primary tumor vs those experiencing a new lesion under 4 cm away.

**Supplementary Figure S4:** Oncoprint of the alterations found in at least five percent of the patients (n=204 pts). X-axis: Patients; Y-axis left side: Percentage of patients with the alteration. Y-axis right side: Alteration name (deep deletions are bi-allelic deletions); (Note: if a patient harbored several alterations in the same gene, only one is visualized). The patient cases are grouped first according to the progression pattern experienced and secondly according to their progression free survival.
